# Supplementary material for: Dynamic spatial coding in parietal cortex mediates tactile-motor transformation
Source: Nat Commun. 2023 Jul 27;14:4532. doi: 10.1038/s41467-023-39959-4 (PMC10374589; doi:10.1038/s41467-023-39959-4)
Supplement: Supplementary file 3 — Reporting Summary [file 41467_2023_39959_MOESM3_ESM.pdf]

nature portfolio

Corresponding author(s): Tobias Heed

Last updated by author(s): May 30, 2023

Reporting Summary

Nature Portfolio wishes to improve the reproducibility of the work that we publish. This form provides structure for consistency and transparency in reporting. For further information on Nature Portfolio policies, see our Editorial Policies and the Editorial Policy Checklist.

Statistics

For all statistical analyses, confirm that the following items are present in the figure legend, table legend, main text, or Methods section.

n/a	Confirmed	
☐	☒	The exact sample size (n) for each experimental group/condition, given as a discrete number and unit of measurement
☐	☒	A statement on whether measurements were taken from distinct samples or whether the same sample was measured repeatedly
☐	☒	The statistical test(s) used AND whether they are one- or two-sided<br>*Only common tests should be described solely by name; describe more complex techniques in the Methods section.*
☐	☒	A description of all covariates tested
☐	☒	A description of any assumptions or corrections, such as tests of normality and adjustment for multiple comparisons
☐	☒	A full description of the statistical parameters including central tendency (e.g. means) or other basic estimates (e.g. regression coefficient) AND variation (e.g. standard deviation) or associated estimates of uncertainty (e.g. confidence intervals)
☐	☒	For null hypothesis testing, the test statistic (e.g. F, t, r) with confidence intervals, effect sizes, degrees of freedom and P value noted<br>*Give P values as exact values whenever suitable.*
☒	☐	For Bayesian analysis, information on the choice of priors and Markov chain Monte Carlo settings
☐	☒	For hierarchical and complex designs, identification of the appropriate level for tests and full reporting of outcomes
☒	☐	Estimates of effect sizes (e.g. Cohen's d, Pearson's r), indicating how they were calculated

Our web collection on statistics for biologists contains articles on many of the points above.

Software and code

Policy information about availability of computer code

Data collection	Experimental protocols were controlled via the software Presentation, version 17.0 (Neurobehavioral Systems, Albany, USA).
Data analysis	fMRI data analysis was conducted using MATLAB R2015a (The MathWorks, Natick, USA), with the following toolboxes: Statistical Parametric Mapping (SPM12, Statistical Parametric Mapping; http://www.fil.ion.ucl.ac.uk/spm/), The Decoding Toolbox version 3.997 (Hebart et al., 2015) and the Statistical nonParametric Mapping toolbox (SnPM13 http://www.nisox.org/Software/SnPM13/). MVPA classification was conducted using the LIBSVM classifier (Chang & Lin, 2011). Whole-brain data visualization was conducted using the Computerized Anatomical Reconstruction and Editing Toolkit (Caret), Pysurfer (https://pysurfer.github.io/) and Freesurfer (https://surfer.nmr.mgh.harvard.edu/). For regions of interest, group mean, individual means and bootstrapped confidence intervals of decoding accuracy were calculated with the Hmisc package (Harrell, 2018) in R and visualized using ggplot2 (Wickham, 2009). Conversion of EyeLink Edf files was done with the Edf2Mat Matlab Toolbox. Custom code developed for the study is available at: https://doi.org/10.17605/OSF.IO/5BN2V

For manuscripts utilizing custom algorithms or software that are central to the research but not yet described in published literature, software must be made available to editors and reviewers. We strongly encourage code deposition in a community repository (e.g. GitHub). See the Nature Portfolio guidelines for submitting code & software for further information.

Data

Policy information about availability of data

All manuscripts must include a data availability statement. This statement should provide the following information, where applicable:

- Accession codes, unique identifiers, or web links for publicly available datasets
- A description of any restrictions on data availability
- For clinical datasets or third party data, please ensure that the statement adheres to our policy

Raw data cannot be shared due to subject confidentiality requirements. Processed data is provided at https://doi.org/10.17605/OSF.IO/5BN2V . The Conte-69 atlas is available as part of the Caret software package available at: https://sites.wustl.edu/vanessenlab/resources/ .

Human research participants

Policy information about studies involving human research participants and Sex and Gender in Research.

Reporting on sex and gender	Participants of both sexes were included in the present study, and participant sex was determined based on self-reporting. Sex and/or gender based analyses were not performed in the current study, as they were not relevant to the aims of the study.
Population characteristics	See below.
Recruitment	Participants were students of the University of Hamburg. The study was advertised online via the SONA recruitment system. After signing up voluntarily, participants were screened to be eligible for the study, particularly regarding any indications that they should not enter an MR environment. This check was done by personnel of the MR facility.<br>As participants signed up out of their own motivation, there may be self-selection biases, e.g. regarding the willingness to participate in several scan sessions, willingness to enter an MR machine etc. We do not think that such biases would affect the results of our sensorimotor-related study.
Ethics oversight	The experiment was approved by the ethics committee of the German Association of Psychology (Deutsche Gesellschaft für Psychologie, DGPs, TB 102011 and TB 102011_add_092014).

Note that full information on the approval of the study protocol must also be provided in the manuscript.

Field-specific reporting

Please select the one below that is the best fit for your research. If you are not sure, read the appropriate sections before making your selection.

- [ ] Life sciences
- [x] Behavioural & social sciences
- [ ] Ecological, evolutionary & environmental sciences

For a reference copy of the document with all sections, see nature.com/documents/nr-reporting-summary-flat.pdf

Behavioural & social sciences study design

All studies must disclose on these points even when the disclosure is negative.

Study description	fMRI study investigating the coding of tactile stimuli relative to the skin vs. external space, as well as the planning of reaching movements to these tactile stimuli. Data are quantitative experimental.
Research sample	The analyzed sample consisted of 16 students of the University of Hamburg (11 female), mean age 23.8 years (range: 19-30 years). Participants were right-handed according to questionnaire-guided self-report (Oldfield, 1971), had normal or corrected-to-normal vision, and reported to be free of any neurological disorders, movement restrictions, or tactile sensitivity problems. Participants provided written informed consent and received course credit or € 8/hour for their participation
Sampling strategy	Participants were recruited via the university's online study portal. We ensured that they were 18-40 years old, did not report any neurological disorders, tactile or visual impairments, and did not take medication that might affect performance (e.g. through alteration of attention etc.).<br>The sample is representative of a student population but may not be representative of the German, European, or world population. Given the N, representativeness may be impossible to achieve. The rationale for our sampling strategy was to test persons aged 18-40 because it has been our experience that older participants sometimes have difficulty following the task instructions, especially in the pace of our experiments. MR recordings are costly and it is important to restrict data acquisition times to a minimum. Therefore, we chose not to slow the pace of our experiment but excluded older participants instead.
Data collection	MRI data was collected with a 3-tesla MR scanner (Siemens, Erlangen) and 32-channel head coil. We monitored the right eye's fixation with an fMRI-compatible eye tracker (Eye Link; SR Research, Ottawa, Canada) operated at a frame rate of 250 Hz. We recorded finger movements by detecting an IR LED on the participant's finger using an IR-sensitive video camera operated at a frame

	rate of 40 Hz. The researcher was not blind to the hypothesis of the study. During the recordings, expert personnel trained to run the MR machine was present (1 person at all times).
Timing	Data collection began on 17/10/2014 and stopped on 08/06/2015.
Data exclusions	Four further participants, in addition to our analyzed sample, were excluded from data analysis: one had performed >99% of all movements to the incorrect movement goal in the crossed, but not in the uncrossed foot posture, MR slice positioning accidentally omitted part of SPL for two participants, and only partial data was collected due to a technical error for one pilot participant.
Non-participation	No participants dropped out/declined participation.
Randomization	Participants were not allocated into experimental groups.

Reporting for specific materials, systems and methods

We require information from authors about some types of materials, experimental systems and methods used in many studies. Here, indicate whether each material, system or method listed is relevant to your study. If you are not sure if a list item applies to your research, read the appropriate section before selecting a response.

Materials & experimental systems

n/a	Involved in the study
☒	☐ Antibodies
☒	☐ Eukaryotic cell lines
☒	☐ Palaeontology and archaeology
☒	☐ Animals and other organisms
☒	☐ Clinical data
☒	☐ Dual use research of concern

Methods

n/a	Involved in the study
☒	☐ ChIP-seq
☒	☐ Flow cytometry
☐	☒ MRI-based neuroimaging

Magnetic resonance imaging

Experimental design

Design type	Task, event-related design.
Design specifications	Each participant completed two MRI sessions on different days and a session where they practiced the task in a regular lab a few days before the first MRI session. In each MRI session, participants completed 3 runs with legs uncrossed and 3 runs with legs crossed. Each run contained 33 trials. Each trial lasted between 7.52 s and 18.8 s, with variation due to inter-trial jitter of stimulus onset and movement instruction cue. The next trial began with a 1.88 s fixation interval, 1.88 s after the movement execution cue was presented.
Behavioral performance measures	Participants finger movements were recorded by tracking an IR LED on the participant's finger using an IR-sensitive video camera. We extracted finger position and movement direction (left vs. right, as seen from the participant's viewpoint) from the video with a custom-made, semi-automated procedure that was based on a combination of cluster detection methods, gradual averaging, and subtraction of images and automatically detected the changes of finger position across frames. Task accuracy was assessed by the mean percentage of movements made in the correction direction of the target. Participants eye position was monitored using an eye tracker to ensure they kept fixation throughout the trial. Error trials were defined as trials where saccade eye movements were detected. These were defined as a 2 s.d. deviation from the trial's mean eye position that also exceeded 20 pixels (0.5° of visual angle) on the presentation monitor.

Acquisition

Imaging type(s)	functional, structural
Field strength	3 Tesla
Sequence & imaging parameters	fMRI: echo planar imaging (EPI) T2*-sensitive sequence that acquired 32 axial slices in descending order (in-plane voxel size: 3 x 3 mm; slice thickness: 3 mm; slice gap: 0.51 mm; TR: 1880 ms; TE: 30 ms; flip angle: 70°; FOV: 216 x 216 mm). Structural MRI: T1* sensitive MPRAGE sequence with 240 slices, 1 x 1 x 1 mm.
Area of acquisition	Whole brain

Diffusion MRI ☐ Used ☒ Not used

Preprocessing

Preprocessing software	Preprocessing was conducted using the software Statistical Parametric Mapping (SPM12, Statistical Parametric Mapping;

	http://www.fil.ion.ucl.ac.uk/spm/), integrated into MATLAB R2015a (The MathWorks, Natick, USA). We corrected fMRI data for susceptibility artifacts and rigid body motion by unwarping and alignment to the first image of the first session. Then, we corrected functional images for differences in acquisition time, and co-registered the individual T1 image to the mean functional image generated during realignment.
Normalization	MVPA classification analyses were performed in subject-space. The resulting whole-brain classification accuracy maps were then normalized to MNI space based on the transformation parameters obtained during segmentation of the T1 image, using non-linear normalization. We then applied 6 mm Gaussian kernel smoothing.
Normalization template	SPM tissue probability map (TPM.nii), in MNI space
Noise and artifact removal	We accounted for baseline drifts within runs by applying a high-pass filter (128 s), and for serial dependency within runs by using an autoregressive model.
Volume censoring	We did not perform volume censoring.

Statistical modeling & inference

Model type and settings

Multivariate modeling was used. First-level GLMs for each participant included 23 predictors that modeled experimentally induced variance of the measured BOLD signal in each voxel with delta functions marking the onsets of the particular delay, convolved, in turn, with the canonical hemodynamic response function. There were 2 baseline predictors, one for each foot posture (uncrossed, crossed) that modeled fixation delays at the beginning of each trial, as well as during the rest periods at the beginning and end of each run. Four predictors modeled the touch localization delay (uncrossed, crossed foot posture x stimulation of left, right foot). Eight predictors modeled the planning and movement execution delays (uncrossed, crossed foot posture x stimulation of left, right foot x pro-, anti-movement). Finally, all trial phases that contained behavioral errors were assigned to a common predictor. Recall, that foot posture was either uncrossed or crossed in a given run. Therefore, any given run contained only 12 (1 baseline, 2 tactile locations, 4 planning, 4 execution, 1 error) of the 23 experiment-wide predictors.

MVPA classification was performed using The Decoding Toolbox on the β-images for the different trial phases and experimental conditions estimated by the GLM. Second-level models, conducted using the SnPM Toolbox, tested for higher-than-chance classification accuracy across the whole brain.

Effect(s) tested

Second-level analyses tested for higher-than-chance classification of left vs. right anatomical tactile stimulus location, external-spatial tactile stimulus location and movement goal location, as well as pro- vs. anti-pointing movement task. One-sample t-tests were performed for each classification.

Specify type of analysis: ☒ Whole brain ☐ ROI-based ☐ Both

Statistic type for inference (See Eklund et al. 2016)

Group analyses tested which brain voxels contained accuracy values that differed significantly from chance-level (50%) using a one-sample t-test. Significance was determined using whole-brain cluster-based permutation tests using an initial threshold of $p<.001$ and a secondary family-wise error (FWE) correction rate of $p<.05$.

Correction

Family-wise error (FWE) correction rate of $p<.05$.

Models & analysis

n/a	Involved in the study
☒	☐ Functional and/or effective connectivity
☒	☐ Graph analysis
☐	☒ Multivariate modeling or predictive analysis

Multivariate modeling and predictive analysis

We used an L2-norm support vector machine (SVM) as classifier in the implementation of LIBSVM, with a fixed cost of $c = 1$. For whole-brain unbiased voxel selection, we applied a spherical searchlight with a radius of 4 voxels. On each classification fold, the classifier was trained with input patterns from run-wise β-images to differentiate between two classes, such as movement goal left vs. movement goal right. Classifier performance was validated with a leave-one-out cross validation design that predicted each of the 12 runs after training based on the respective 11 other runs. We report the mean decoding accuracy across all classification iterations, depicted at the center voxel of a given searchlight region, as the measure of the overall generalization performance of the classifier of that searchlight region. We repeated this procedure for all recorded voxels, resulting in a whole-brain map of averaged decoding accuracy for the two tested conditions across all test runs.
